# Lymphangiosis carcinomatosa in squamous cell carcinomas of larynx and hypopharynx – value of conventional evaluation and additional immunohistochemical staining of D2-40

**DOI:** 10.1186/1477-7819-7-25

**Published:** 2009-03-04

**Authors:** Hans-Ullrich Völker, Matthias Scheich, Isabell Nowack, Alexandra Metzger, Imme Haubitz, Bernhard Puppe, Rudolf Hagen, Hans-Konrad Müller-Hermelink, Christiane Völter

**Affiliations:** 1 Institute of Pathology, University of Würzburg, Germany; 2 Dept. of Otorhinolaryngology, University of Würzburg, Germany

## Abstract

**Background:**

Recent studies revealed a predictive value of lymphatic vessel invasion (L1) for the nodal metastasizing and poor prognosis in malignant tumors at different sites. The monoclonal antibody D2-40 (podoplanin) stains specifically endothelial cells of lymphatic vessels and improves the search for L1. However, the importance of this immunohistochemical staining was not investigated in squamous cell carcinomas (SCC) of larynx and hypopharynx.

**Aim:**

This study was performed to compare the diagnostic potential of convential and immunohistochemical determination of L1 in SCC of larynx and hypopharynx with special respect to the predictive value for nodal metastasizing and prognosis.

**Methods:**

119 SCCs of the larynx (n = 70) respectively hypopharynx (n = 49) were investigated. The lymphatic vessel invasion was assessed by conventional method (HE stain) and immunohistochemical staining with an antibody against D2-40 (DAKO, Germany). Immunohistochemistry was performed in accordance with manufacturer's protocol. L1 was searched microscopically in a standardized magnification (×200) in serial sections of tumor samples (1 section per cm tumor diameter).

**Results:**

The immunohistochemical investigation did not show significant advantages for the prediction of regional nodal metastases. Despite a low sensitivity (< 50%) in both methods, the specifity can reach 80%. The negative predictive value in both methods seems acceptable (up to 80%), whereas the positive predictive value is not higher than 64%. Cases with L1 detected either conventionally or immunohistochemically did not show a significant shorter survival than cases with L0. However, a non-significant shorter survival was found. Only in SCC of hypopharynx, a combination of both methods revealed patients with a significant worse prognosis.

**Conclusion:**

The status of lymphatic vessel invasion should be documented in standardized tumor reports. A benefit of an additional immunohistochemical investigation was not found, for the daily routine HE-stain seems sufficient.

## Background

Squamous cell carcinomas (SCC) are the most common malignant tumors of the larynx and hypopharynx. The tumor morphology does not differ at both sites, but a different behavior in nodal metastasizing is well known. Whereas glottic laryngeal SCC metastasize late and in a lower number, SCC of hypopharynx may develope metastases early in the course of disease. Own investigations revealed metastases in up to 41% of a series of glottic SCC and up to 92% of carcinomas of hypopharynx (tumor stages > pT1) [1]. Apart from statistical considerations, tumors at both sites can either metastasize in a early stage or be free of metastases even in advanced stages.

The extent of surgery regarding the neck dissection (ND) depends on the clinical stage of nodal involvement. A problem are occult nodal metastases or micrometastases. Up to 30% of cN0 cases show metastases in histological investigations [2].

A better prediction of metastatic potential could influence therapeutical approaches – in cases with low risk extended surgery with (complete) neck dissection could be avoided and replaced e.g. by local clinical controls [3].

Some efforts have been undertaken for finding predictive parameters, up to now without substantive success [1]. In particular parameters obtainable in the routine histomorphological investigation seem attractive. One point of interest is the importance of lymphatic vessel invasion (L1 according with the TNM classification of UICC). From a mechanistical point of view, it could be an important condition for development of nodal metastases. A higher degree of lymphangiogenesis and lymphatic vessel density is associated with increasing frequency of nodal metastases [4-7]. But the lymphatic vessel density is difficult to graduate in the daily work, whereas the assessment of lymphatic vessel invasion is more easy. However, most of recently published studies have not found a significant correlation of conventional determination of L1 in HE-stain (hematoxylin-eosin) with nodal metastasizing. Currently, for some malignant tumors (e.g. breast carcinoma, carcinoma of uterine cervix, or esophageal carcinoma) a significant relation between L1 and N+ was reported using immunohistochemical methods for the assessment of L1 [8-10].

The monoclonal antibody D2-40 (podoplanin) recognizes a fixation-resistant O-linked sialoglycoprotein epitope on lymphatic endothelium. Endothelial cells of blood vessels remain negative in this staining [11,12]. To our knowlegde, no study is published, which investigated the value of an additional immunohistochemical analysis looking for lymphatic vessel invasion in SCC of the larynx or hypopharynx.

Therefore, we performed this study to evaluate the diagnostic value of immunohistochemistry in the assessment of lymphatic vessel invasion in SCC of larynx and hypopharynx with special respect to predict the risk of nodal metastases and individual prognosis.

## Materials and methods

**Figure 1:**
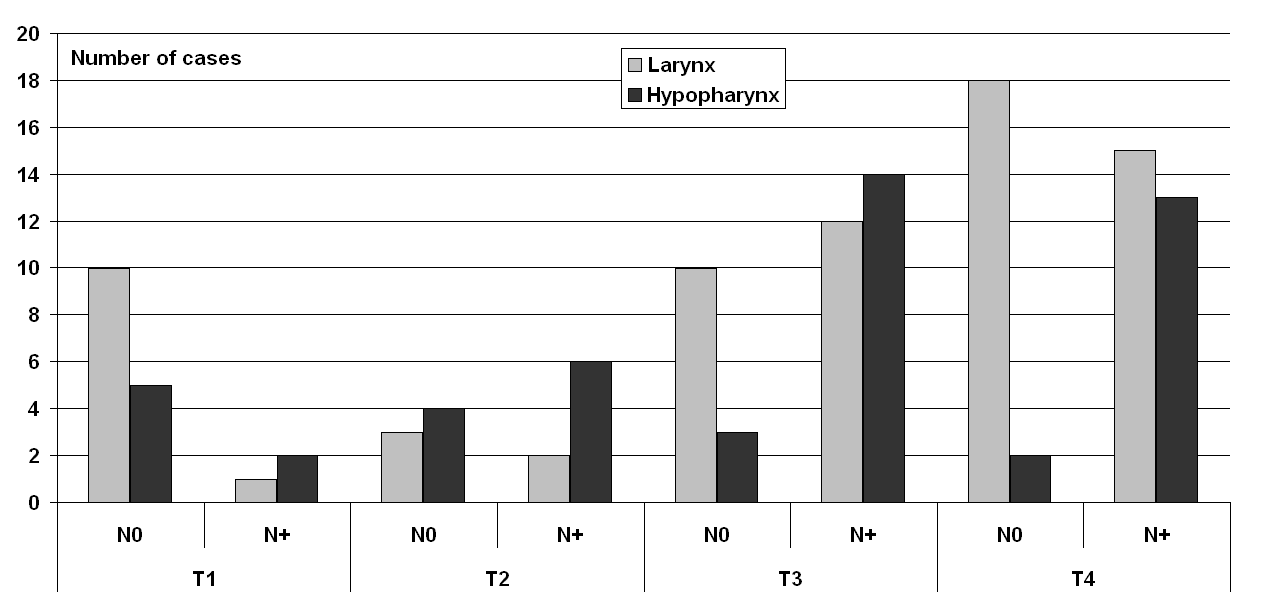
**Distribution of cases in different tumor stages**.

119 cases (between 1996 up to 2002, follow up at least 5 years) with 70 SCC of glottic larynx and 49 SCC of hypopharynx were determined. Complete clinical data of all patients were available. Table 1 shows the distribution of cases and main clinical characteristics, Figure 1 indicates the distribution of cases in several tumor stages accordingly to TNM classification for malignant tumors of UICC. All patients included in this study were treated by complete laryngectomy with ND, with exception of laryngeal pT1 tumors, in which metastasizing was excluded by clinical investigations (e.g. ultrasound). The latter were treated by local tumor excision (hemilaryngectomy). Apart from these cases, all others received a postoperative radiotherapy.

**Table 1:** Main characteristics of cases included in the study.

	**Larynx n = 70**	**Hypopharynx n = 49**
**Median Age [Years]**	62.2 (45–85)	57.2 (38–75)*

**Male:Female**	66:4	43:6

**Median tumor size [cm]**	2.5 (0.6–5.2)	3.4 (1.0–6.5)*

**Grading (G1/G2/G3)**	5/43/22	1/25/23

**Median number of investigated lymph nodes**	30.4 (4–76)	31.5 (5–82)

**N0/N+**	30/40 (43% N+)	35/14 (71% N+)*

**Median number of nodal metastases in N+**	2 (1–25)	3 (1–17)

* P < 0.05

Only cases with well preserved tumor specimen (no autolysis, well formalin-fixed, no mechanical or thermical alterations) were included. One tumor sample per cm tumor diameter was investigated.

For standard staining and immunohistochemistry serially prepared sections were examined. In large tumors, especially areas of the lateral and deep tumor borders (front of invasion) were investigated.

The histomorphological diagnoses of SCC and its grading was reevaluated by an experienced pathologist. The resected lymph nodes were also reevaluated.

Immunohistochemistry was performed in a standard procedure of daily routine. Briefly, sections (2–5 μm) for immunohistochemistry were air-dried overnight (at least 12 hours), dewaxed, rehydrated in descending concentrations of ethanol before being heated for antigen unmasking in 10 mM citric acid (pH 5.5) for five minutes. After rinsing with distilled water, slides were washed in phosphate buffered saline (PBS). For staining, the ADVANCE kit (DAKO, Germany) was used in accordance to the manufacturer's protocol. The antibody for D2-40 (clone D2-40, DAKO, Germany) was used in a dilution of 1:800.

**Figure 2:**
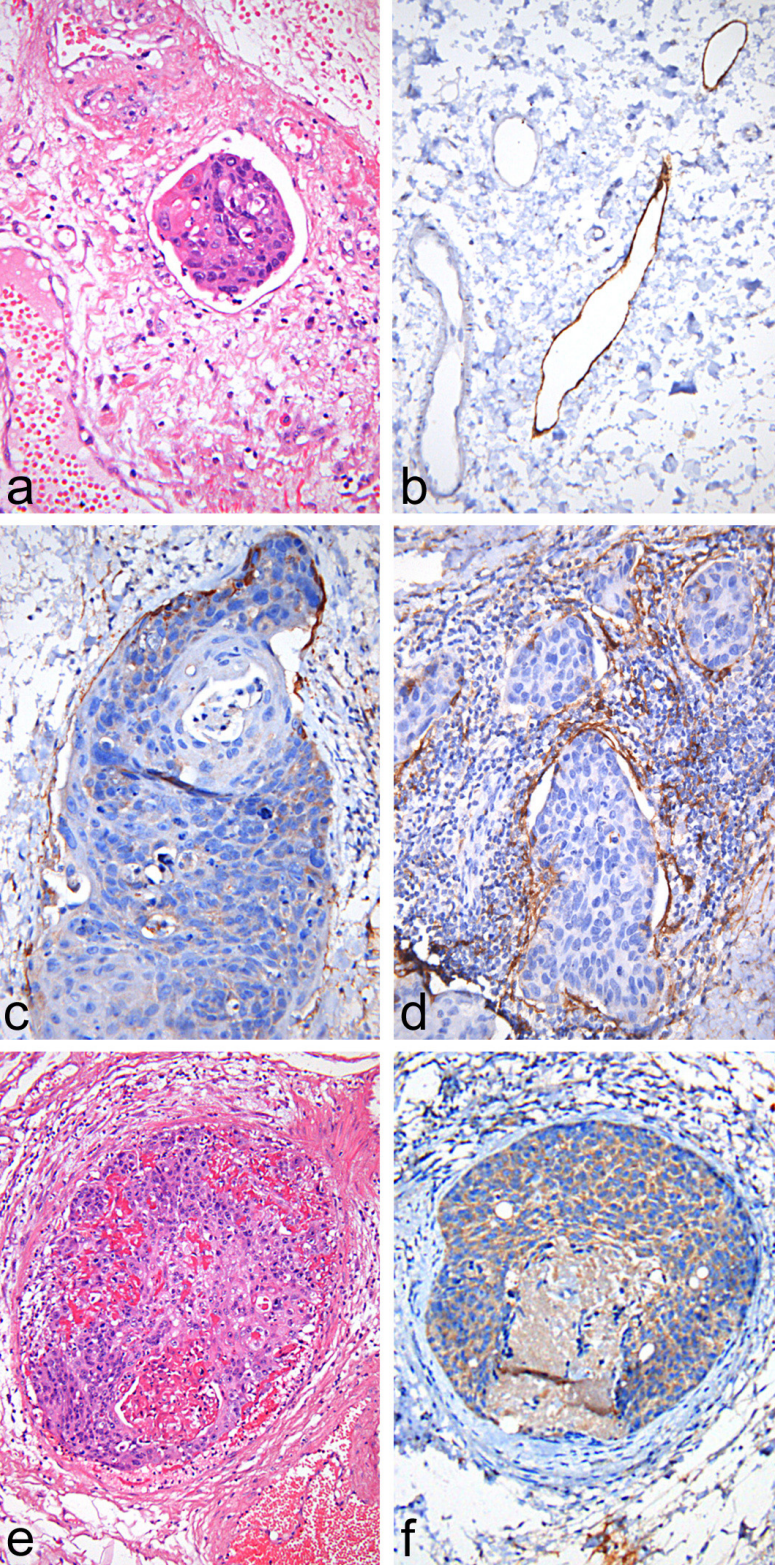
**a) Lymphangiosis carcinomatosa in conventional staining (HE ×400); b) Specific reaction in lymphatic vessel endothelium (right) and no reaction in a blood capillary (left) (D2-40 ×400); c) and d) Different types of lymphatic vessel invasion: in c) single larger vessel, in d) some small vessels surrounded by inflammatory response (D2-40 ×400); e) tumor thromembolism in a blood vessel (note: erythrocytes) (HE ×400) – f) negative for D2-40 (D2-40 ×400)**.

All slides were determined microscopically with standardized magnification (×200). Only undoubted lymphatic vessel invasion was assessed positive (L1) in conventional (HE) stain. This means, L1 was diagnosed in cases with tumor embolism in morphologically clear lymphatic vessels with identifiable endothels and thin vessel wall, an example is given in Figure 2a. For immunohistochemical analysis of L1, all samples contained an internal positive control of staining in tumor-free lymphatic vessels (Figure 2b). Tumor thrombembolism in vessels with stained endothelium in immunoperoxidase was the criterion for L1 in immunohistochemical analysis (Figure 2c, d). The number or location of invaded vessels was not respected, because the biological value of differences between one or more invaded vessels remains unclear and a cut-off is not definable. The different density of lymphatic vessels in larynx and hypopharynx was not considered, but cases without peritumorous lymphatic vessels were excluded before starting the study. Definite invading of non-lymphatic vessels (tumorembolism with fibrinous reaction, broad muscular wall of the vessel, or negativity in immunoperoxidase (Figure 2d, e)) were excluded.

For survival analysis, the overall survival was considered. The disease-free survival was not respected. Only synchronous nodal metastases at time of primary diagnosis were included as N+. Distant metastases (e.g. lung, liver) out of regional lymph nodes were not considered, because their appearance is rather a result of invasion of blood vessels than lymphatic vessels.

L-status L0/L1 (with both methods) was correlated with nodal involvement, stage and size of the tumor, and survival. Sensitivity and specificity were calculated. Data were processed by Microsoft Excel and SPSS for descriptive statistic values, Student's t-test, Chi-square test, U-test by Mann-Whitney, or log rank test. Data were analyzed for both separated groups (larynx, hypopharynx) and overall.

## Results

Tumor size (P < 0.001), tumor stage T (larynx P < 0.001; hypopharynx P < 0.05), and grading G (larynx P < 0.001; hypopharynx P < 0.05) were significant higher in metastasized SCC at both anatomic sites.

Overall (n = 119), 37.8% of tumors showed L1 within the conventional assessment, and 35.3% using the immunohistochemical method. 50.4% showed L1 either conventionally or immunohistochemically. In 32.7% a discrepancy regarding L1 status was found between both methods, 26.3% of L1 cases in immunohistochemistry were conventionally negative (L0). The reliablity for nonconformance was kappa = 0.27.

**Figure 3:**
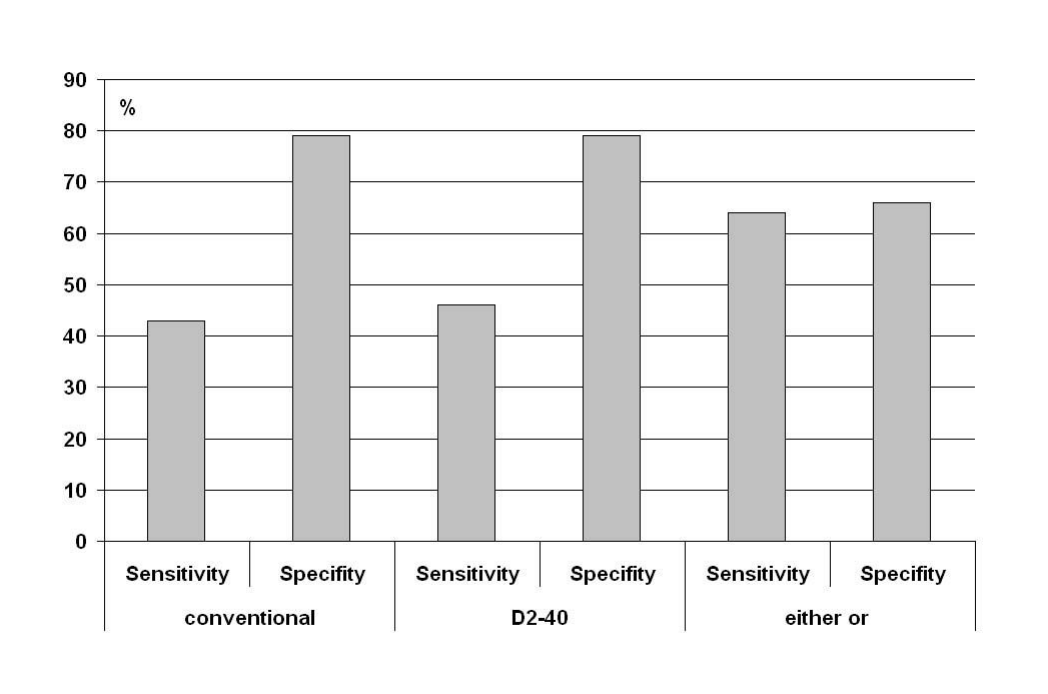
**Sensitivity and specifity of the different methods for evaluation of lymphatic vessel invasion concerning the status of nodal metastasizing**.

The results in the different groups are indicated in table 2. The sensitivity and specificity of L1 regarding the predictive value of nodal metastasizing was low (Figure 3).

**Table 2:** Results of assessment of L-status conventionally and immunohistochemically in both groups.

		**Larynx (n = 70)**		**Hypopharynx (n = 49)**		
		**n**	**%**	**n**	**%**	**P (Chi-square)**

**conventional**	**negative**	51	72.86%	29	59.18%	0.12
		
	**positive**	19	27.14%	20	40.82%	

**D2-40**	**negative**	45	64.29%	32	65.31%	0.91
		
	**positive**	25	35.71%	17	34.69%	

**either or**	**negative**	36	51.43%	23	46.94%	0.63
		
	**positive**	34	48.57%	26	53.06%	

**discrepancy**	**no**	46	65.71%	34	69.39%	0.67
		
	**yes**	24	34.29%	15	30.61%	

**D2-40**	**positive in conventionally L0**	15	21.43%	6	12.24%	0.19

The correlation of L1 and nodal involvement revealed a significant better correlation between L1 (conventionally or immunohistochemically) with N+ in cases with more than one nodal metastasis (P < 0.05). The other results are given in table 3.

**Table 3:** L-status and nodal involvement. Positive and negative predictive values.

		**N0**		**N+**		
		**n**	**%**	**n**	**%**	**P (Chi-square**

**conventional**	**L0**	43	79.63%	37	56.92%	0.0077
		
	**L1**	11	20.37%	28	43.08%	

**D2-40**	**L0**	42	77.78%	35	53.85%	0.0059
		
	**L1**	12	22.22%	30	46.15%	

**either or**	**L0**	36	66.67%	23	35.38%	0.00061
		
	**L1**	18	33.33%	42	64.62%	

**Figure 4:**
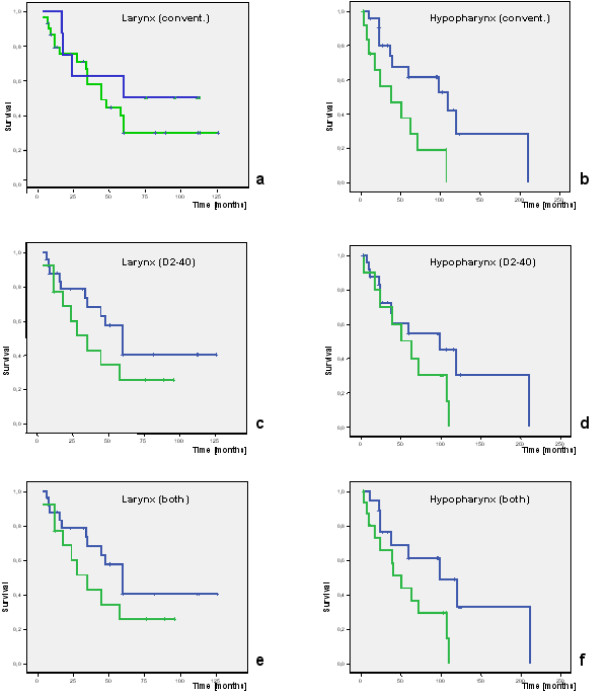
**Survival (Kaplan-Meier) for SCC with L0 (blue) and L1 (green) for the different methods of investigation: a) + b) conventional; P = 0.4 resp. P = 0.01. c) + d) D2-40 immunohistochemistry; P = 0.3 resp. P = 0.17 e) + f) both methods together; P = 0.2 resp. P = 0.049 (log rank)**.

In survival analysis, parameters apart from L-status showed a significant shorter survival with increasing tumor stage T (P = 0.038) and N+ (P = 0.0048, also significant in multivariate analysis). The L-status (L0/L1) did not influence the survival significantly, even though a lymphatic vessel invasion was accompanied by a shorter survival (figure 4). However, the results were better for hypopharyngeal SCC, the combination of both methods showed significant differences between L0 and L1 (P = 0.049, log rank test), independent of nodal status.

## Discussion

This study was performed to assess the predictive value for nodal metastasizing of lymphatic vessel invasion (L1) using the conventional and immunohistochemical method in squamous cell carcinomas (SCC) of larynx and hypopharynx. The used antibody D2-40 (podoplanin) recognizes specifically endothelium of lymphatic vessels, whereas blood vessels remain negative [12,13].

With the background of unknown individual potential for metastasising, this study confirmed data of conventional assessment of L1, which argues against a significant predictive value of this investigation [1]. However, apart from metastases greater than 2 mm, micrometastases are possible. Different authors have described a prognostic impact of detecting micrometastases [14]. We did not search for micrometastasis < 1 mm with immunohistochemical methods (e.g. Pan-Keratin AE1/3) in this study, but small metastases with 1–2 mm were found in single cases and staged as N+. Nevertheless, the importance of L1 for the development of micrometastasis < 1 mm should be evaluated in further studies.

The negative predictive value reached up to 80%, whereas a positive prediction is not possible. The reason is the discrepance between the development of lymphatic vessel invasion in nodal negative cases, which may develope the metastases later in the course of disease.

The decision between lymphatic and blood vessel invasion shows a high interobserver variability, because the morphological discrimination between small lymphatic and blood vessels is difficult. The question was, whether the immunohistochemical detection of lymphatic vessles can improve the predictive value of this investigation. However, the specifity and sensitivity was not better with support of immunohistochemical staining. This results did not confirm the reported advantages of D2-40 staining in other tumors [8,15,16]. Another item is the importance of lymphatic vessel density [4,5,7,17], whose investigation was not the aim of this study. Some authors found a higher potential for metastasizing in tumors with increased density of lymphatic vessels, however, the measurement of vessel density does not seem practicable in the daily routine.

The immunohistochemical investigation seems at first appearance a convenient and reliable method. Some cases negative in conventional investigation showed L1 in immunohistochemistry. However, several cases positive for L1 conventionally became negative in immunohistochemistry. One reason could be the fragile endothelial layer in lymphatic vessels, which can be destroyed by thrombembolism of tumor cells. The presence of the endothelium is needed for the success of this analysis. Another reason is, that the conventional assessment cannot distinguish between lymphatic and blood vessels (apart from cases with typical pictures of blood vessel invasion, e.g. embolism with degenerated erythrocytes and fibrin precipitation). In these cases, a positive result of L1 could be false positive and the correct classification would be V1 (venous invasion). With regard of this point, the immunohistochemical assesment has some advantages, however, they are not important for the predictive potential of this investigation.

Our investigations were not suitable to explain, why laryngeal carcinomas show a lower potential for nodal metastasizing (including advanced tumor stages) than hypopharyngeal SCC.

## Conclusion – clinical applicability

It seems not possible to predict the nodal metastasizing of laryngeal or hypopharyngeal SCCs with the status of lymphatic vessel invasion. The immunohistochemical detection is not helpful in the daily routine. Nodal metastasizing was not found in 33% of tumors showing L1 defined from the results of either methods. The value is higher than that from the results of only one examination, standard or immunohistochemical staining. From these results, both methods together have little value for the prediction of N+. However, the negative predictive value is acceptable.

Recommendations regarding the necessity of neck dissection (ND) in cN0 cases are difficult, however, in borderline cases the (modified) ND should be performed in tumors with L1. In L0 cases with no other clinical arguments for ND, the renouncement of ND flanked by regular clinical controls seems acceptable.

Despite the not-significant shorter survival of patients with L1 in comparison with patients with L0, the status of lymphatic vessel invasion (L0/L1) should mandatory be reported in histopathological diagnoses for a standardized tumor documentation.

## Competing interests

The authors declare that they have no competing interests.

## Authors' contributions

HUV was involved in the idea, histopathology, evaluation of staining, manuscript. IN was involved in the evaluation of staining, results. MS, RH, CV were involved in clinical data and details, discussion. IH, BP were involved in statistical evaluation, results. HKMH, AM were involved in histopathology, discussion.
